# miR-221/222 Target the DNA Methyltransferase MGMT in Glioma Cells

**DOI:** 10.1371/journal.pone.0074466

**Published:** 2013-09-19

**Authors:** Cristina Quintavalle, Davide Mangani, Giuseppina Roscigno, Giulia Romano, Angel Diaz-Lagares, Margherita Iaboni, Elvira Donnarumma, Danilo Fiore, Pasqualino De Marinis, Ylermi Soini, Manel Esteller, Gerolama Condorelli

**Affiliations:** 1 Department of Molecular Medicine and Medical Biotechnology, "Federico II” University ofNaples, Naples, Italy; 2 IEOS, CNR, Naples, Italy; 3 Fondazione IRCCS SDN, Naples, Italy; 4 Epigenetic and Cancer Biology Program (PEBC) IDIBELL, Hospital Duran i Reynals, Barcelona, Spain; 5 Ospedale Cardarelli, Naples, Italy; 6 Department of Pathology and Forensic Medicine, Institute of Clinical Medicine, Pathology and Forensic Medicine, School of Medicine, Cancer Center of Eastern Finland, University of Eastern Finland, Kuopio, Finland; University of California-San Francisco, United States of America

## Abstract

Glioblastoma multiforme (GBM) is one of the most deadly types of cancer. To date, the best clinical approach for treatment is based on administration of temozolomide (TMZ) in combination with radiotherapy. Much evidence suggests that the intracellular level of the alkylating enzyme O^6^-methylguanine–DNA methyltransferase (MGMT) impacts response to TMZ in GBM patients. MGMT expression is regulated by the methylation of its promoter. However, evidence indicates that this is not the only regulatory mechanism present. Here, we describe a hitherto unknown microRNA-mediated mechanism of MGMT expression regulation. We show that miR-221 and miR-222 are upregulated in GMB patients and that these paralogues target MGMT mRNA, inducing greater TMZ-mediated cell death. However, miR-221/miR-222 also increase DNA damage and, thus, chromosomal rearrangements. Indeed, miR-221 overexpression in glioma cells led to an increase in markers of DNA damage, an effect rescued by re-expression of MGMT. Thus, chronic miR-221/222-mediated MGMT downregulation may render cells unable to repair genetic damage. This, associated also to miR-221/222 oncogenic potential, may poor GBM prognosis.

## Introduction

Glioblastoma multiforme (GBM) is the most common and deadly primary tumor of the central nervous system. Despite several therapeutic advances, the prognosis for GBM remains poor, with a median survival lower than 15 months [1,2]. Currently, first-line therapy for GBM comprises surgery with the maximum feasible resection, followed by a combination of radiotherapy and treatment with the alkylating agent temozolomide (TMZ), also referred to by its brand name Temodal [3,4,5]. TMZ is a methylating agent that modifies DNA in several positions, one of them being O^6^-methylguanine MeG (O^6^MeG) [6]. If the methyl group is not removed before cell division, this modified guanine preferentially pairs with thymine during DNA replication, triggering the DNA mismatch repair (MMR) pathway, DNA double-strand breaks, and, therefore, the apoptotic pathway [7,8]. O^6^-methylguanine–methyltrasferase (MGMT) is a suicide cellular DNA repair enzyme ubiquitously expressed in normal human tissues. MGMT does not act as a part of a repair complex but works alone [9]. To neutralize the cytotoxic effects of alkylating agents, such as TMZ, it rapidly reverses alkylation at the O^6^ position of guanine, transferring the alkyl group to an internal cysteine residue in its active site. In this form, the enzyme is inactive and, thus, requires *de novo* protein synthesis. In tumors, high levels of MGMT activity are associated with resistance to alkylating agents [10]. In contrast, epigenetic silencing of MGMT gene expression by promoter methylation results in sensitization to therapy [11,12]. However, some studies have reported that MGMT promoter methylation does not always correlate with MGMT expression and with response to therapy [13,14]. Therefore, the existence of other mechanisms of MGMT regulation should be postulated.

MicroRNAs (miRs) are small regulatory molecules that have a role in cancer progression and in tumor therapy response [15,16]. By negatively regulating the expression of their targets, miRs can act as tumor suppressors or oncogenes [17]. miRs may also regulate DNA damage response and DNA repair, interfering with the response to chemotherapy or radiotherapy [18]. Several studies have indicated that the modulation of miR expression levels is a possible therapeutic strategy for cancer.

The paralogues miR-221 and miR-222 have frequently been found to be dysregulated in glioblastoma and astrocytomas [19,20,21,22]. Their upregulation increases glioma cell proliferation, motility, and *in vivo* growth in mouse models. miR-221/222 have also been shown to be implicated in cellular sensitivity to tumor necrosis factor-related apoptosis-inducing ligand (TRAIL)-treatment [23,24,25]. In this manuscript, we provide evidence that miR-221 and miR-222 regulate MGMT expression levels in glioblastoma, increasing the response to TMZ, but due to their oncogenic potential, affect overall patient survival negatively.

## Materials and Methods

### Cell culture and transfection

U87MG, T98G, LN428, LN308, A172, and HEK-293 cells were grown in DMEM. LN229 were grown in Advanced DMEM (Gibco, Life technologies, Milan, Italy). T98G, U87MG, and LN229 were from ATCC (LG Standards, Milan Italy); LN428, LN308, and A172 were kindly donated by Frank Furnari (La Jolla University). Media were supplemented with 10% heat-inactivated fetal bovine serum (FBS) -5% FBS for LN229 -2 mM L-glutamine, and 100 U/ml penicillin/streptomycin. All media and supplements were from Sigma Aldrich (Milan, Italy). For overexpression of miRs, cells at 50% confluency were transfected using Oligofectamine (Invitrogen, Milan, Italy) and 100nM pre-miR-221 or pre-miR-222, a scrambled miR or anti-miR-221/222 (Applied Biosystems, Milan, Italy). For overexpression of MGMT, cells were transfected using Lipofectamine and Plus Reagent with 4 µg of MGMT cDNA (Origene, Rockville MD USA). Temozolomide was purchased from Sigma Aldrich (Milan, Italy).

### Human Glioma samples

A total of 34 formalin-fixed, paraffin-embedded (FFPE) tissue samples were collected from the archives of the Department of Pathology, University Hospital of Kuopio, Finland. Permission to use the material was obtained from the National Supervisory Authority for Welfare and Health of Finland, and the study was accepted by the ethical committee of the Northern Savo Hospital District, Kuopio, Finland.

### Primary cell cultures

Glioblastoma specimens were obtained as previously described [19]. Samples were mechanically disaggregated, and the lysates grown in DMEM-F12 medium supplemented with 10% FBS, 1% penicillin streptomycin, and 20 ng/ml epidermal growth factor (EGF; Sigma-Aldrich, Milan, Italy). To determine the glial origin of the isolated cells, we stained the cultures for glial fibrillary acidic protein (GFAP), a protein found in glial cells.

### Protein isolation and Western blotting

Cells were washed twice in ice-cold PBS and lysed in JS buffer (50 mM HEPES pH 7.5 containing 150 mM NaCl, 1% Glycerol, 1% Triton X100, 1.5mM MgCl_2_, 5mM EGTA, 1 mM Na _3_VO_4_, and 1X protease inhibitor cocktail). Protein concentration was determined by the Bradford assay (BioRad, Milan, Italy) using bovine serum albumin (BSA) as the standard, and equal amounts of proteins were analyzed by SDS-PAGE (12.5% acrylamide). Gels were electroblotted onto nitrocellulose membranes (GE Healthcare, Milan, Italy). For immunoblot experiments, membranes were blocked for 1 hr with 5% non-fat dry milk in Tris-buffered saline (TBS) containing 0.1% Tween-20, and incubated at 4°C overnight with primary antibody. Detection was performed by peroxidase-conjugated secondary antibodies using the enhanced chemiluminescence system (GE Healthcare, Milan, Italy). Primary antibodies used were: anti-β-actin from Sigma-Aldrich (Milan Italy); anti-caspase-3 and anti-PARP from Santa Cruz Biotechnologies (Santa Cruz, CA, USA), anti-γH2AX from Millipore (Milan, Italy), anti-p53, p^ser15^ p53, and phosphorylated-ATM from Cell Signaling Technology (Milan, Italy).

### RNA extraction and Real-Time PCR

*Cell culture*: Total RNA (microRNA and mRNA) were extracted using Trizol (Invitrogen, Milan, Italy) according to the manufacturer’s protocol.

### Tissue specimens

Total RNA (miRNA and mRNA) from FFPE tissue specimens was extracted using RecoverAll Total Nucleic Acid isolation Kit (Ambion, Life Technologies, Milan, Italy) according to the manufacturer’s protocol. Reverse transcription of total miRNA was performed starting from equal amounts of total RNA/sample (1µg) using miScript reverse Transcription Kit (Qiagen, Milan, Italy), and with SuperScript® III Reverse Transcriptase (Invitrogen, Milan, Italy) for mRNA. Quantitative analysis of MGMT, β-actin (as an internal reference), miR-221, miR-222, and RNU5A (as an internal reference) were performed by RealTime PCR using specific primers (Qiagen, Milan, Italy), miScript SYBR Green PCR Kit (Qiagen, Milan, Italy), and iQ^TM^ SYBR Green Supermix (Bio-Rad, Milan, Italy), respectively. The reaction for detection of mRNAs was performed as follows: 95°C for 15’, 40 cycles of 94°C for 15″, 60°C for 30″, and 72°C for 30″. The reaction for detection of miRNAs was performed as follows: 95°C for 15’, 40 cycles of 94°C for 15″, 55°C for 30″, and 70°C for 30″. All reactions were run in triplicate. The threshold cycle (CT) is defined as the fractional cycle number at which the fluorescence passes the fixed threshold. For relative quantization, the 2^(-ΔCT)^ method was used as previously described [26]. Experiments were carried out in triplicate for each data point, and data analysis was performed by using a Bio-Rad software (Bio-Rad, Milan, Italy).

### Luciferase assay

The 3’ UTR of the human MGMT gene was PCR amplified using the following primers: MGMT-Fw:5’TCTAGAGTATGTGCAGTAGGATGGATG3’; MGMT-Rv: 5’ TCCAGAGCTACAGGTTTCCCTTCC3’, and cloned downstream of the Renilla luciferase stop codon in pGL3 control vector (Promega, Milan, Italy). A deletion was introduced into the miRNA-binding sites with the QuikChange Mutagenesis Kit (Stratagene, La Jolla CA USA) using the following primers: MGMT-mut Fw: 5’ CTATATCCAAAAGGGAAACCTGTAGCTCTTGC 3’. MGMT-mut Rw: 5’- GCAGAGCTACACGTTTCCCTTTTGGATATAG 3’. HEK-293 cells were co-transfected with 1.2µg of plasmid and 400 µg of a Renilla luciferase expression construct, pRL-TK (Promega, Milan, Italy), with Lipofectamine 2000 (Invitrogen, Milan, Italy). Cells were harvested 24 hrs post-transfection and assayed with Dual Luciferase Assay (Promega, Milan, Italy) according to the manufacturer’s instructions. Three independent experiments were performed in triplicate.

### Cell death quantification

Cell viability was evaluated with the CellTiter 96 AQueous One Solution Cell Proliferation Assay (Promega, Milan, Italy) according to the manufacturer’s protocol. Metabolically active cells were detected by adding 20 µL of MTS to each well. After 2 hrs of incubation, the plates were analyzed in a Multilabel Counter (BioTek, Milan, Italy). For caspase-3 inhibition experiments, ZVAD-Fmk was purchase from Calbiochem.

### Comet assay

Alkaline comet assay was performed accordingly to manufacturer’s instructions (Trevigen, Gaithersburg, Maryland, USA). Briefly, 12x10^4^ glioblastoma cell lines were transfected with miRs or MGMT cDNA and then treated with TMZ in 6-well plates. Cells were collected and then combined with LMAgarose. The mixture was applied to Comet slides and kept at 4°C in the dark for 10’. The slides were immersed in pre-chilled lysis buffer for 30 min. The slides were washed and then electrophoresis was carried out. The slides were fixed in 70% ethanol for 5 min and let dry overnight. SYBR green was added and comets were photographed at 100 x microscopes (Carl Zeiss Inc., NY, USA).

### γH2AX flow cytometric analysis

Treated cells were fixed with 2% paraformaldehyde for 1 hr. Fixed cells were permeabilized with 0.1% Triton-X100/PBS for 5 min on ice. Blocking was done in PBS+2% BSA. Anti-phosphorylated H2Ax antibody(Ser139, γH2Ax, Millipore, Milan, Italy) was diluted in PBS and then FITC-conjugated goat anti-mouse antibody (Santa cruz Biotechnology, CA, USA) was used. Cells were analyzed with a Becton Dickinson FACScan flow cytometer.

### Caspase Assay

The assay was performed using the Colorimetric CaspACE^TM^ Assay System, (Promega, Milan, Italy) as reported in the instruction manual. Briefly, T98G cells were transfected with miR-221 and/or MGMT cDNA, plated in 96-well plates, and then treated with 300 µMol of temozolomide or with 10 µMol of ZVAD-Fmk. After treatments, 100 µl caspase-3/-7 reagent was added to each well for 1 hr in the dark. The plates were analyzed in a Multilabel Counter (BioTek, Milan, Italy).

### MGMT Methylation Analysis

DNA methylation status in the CpG island of *MGMT* was established by PCR analysis of bisulfite modified genomic DNA, which induces chemical conversion of unmethylated, but not methylated, cytosine to uracil. DNA was extracted from cell lines using the DNeasy blood and tissue kit (Qiagen, Milan, Italy). DNA (1 µg) was modified with sodium bisulfite using the EZ DNA methylation-gold kit (Zymo Research, CA, USA) according to the manufacturer’s instructions. Methylation-specific polymerase chain reaction (MSP) was performed with primers specific for either methylated or the modified unmethylated DNA. Primer sequences for the unmethylated reaction were 5'TTTGTGTTTTGATGTTTGTAGGTTTTTGT3' (forward primer) and 5'AACTCCACACTCTTCCAAAAACAAAACA3' (reverse primer), and for the methylated reaction they were 5'TTTCGACGTTCGTAGGTTTTCGC3' (forward primer) and 5'GCACTCTTCCGAAAACGAAACG3' (reverse primer.) The annealing temperature was 59°C. The cell line SW48 and *in vitro* methylated DNA (CpGenome Universal Methylated DNA, Millipore) were used as a positive control for the methylation of MGMT and DNA from normal lymphocytes used as a negative control. Controls without DNA were used for each set of methylation-specific PCR assays. The methylation-specific PCR product was loaded directly onto 2% agarose gels, stained with syber safe, and examined under ultraviolet illumination.

### Colony Assay

Cells were transfected with scrambled miR or miR-221 for 24 hrs, harvested, and 2.4 x10^4^ cells plated in 6-well plates. After 24 hrs, cells were treated with 300 µMol TMZ for 24 hrs, as indicated. Cells were transferred to 100-mm dishes and grown for 6 days. Finally, the cells were colored with 0.1% crystal violet dissolved in 25% methanol for 20 min at 4°C. Dishes were washed with water, left to dry on the bench, and then photographs taken.

### Statistical analysis

Student’s *t* test and nonparametric Mann-Whitney tests were used to determine differences between values for normally and, respectively, not normally distributed variables. A probability level <0.05 was considered significant throughout the analysis. Data were analyzed with GraphPad Prism (San Diego, CA, USA) for Windows.

## Results

### Sensitivity of human glioma cell lines to temozolomide

**Figure 1:**
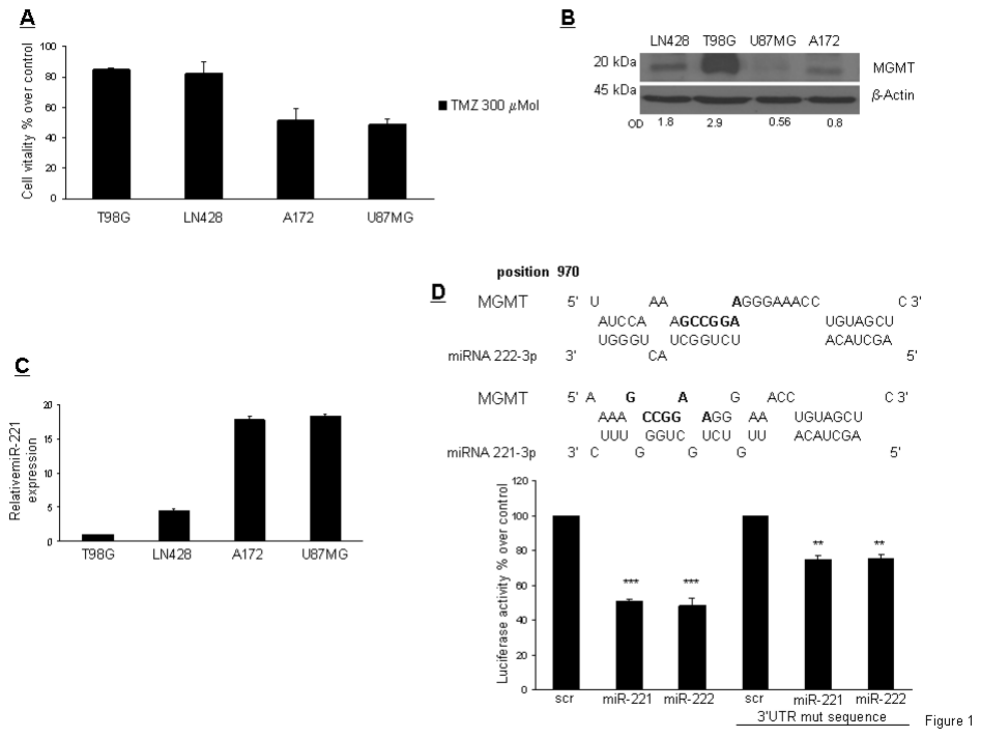
TMZ sensitivity and MGMT and miR-221/222 expression in glioma cells. (A) Glioma cells were treated with TMZ (300µMol) for 24 hr. Cell viability was evaluated with an MTT assay. (B) Western blot analysis of MGMT expression in glioblastoma cells. (C) Real time PCR of miR-221 expression in glioblastoma cells. (D) RNA Hybrid prediction analyzes of miR-222, miR-221, and MGMT 3’ UTR. In bold are shown the mutated oligonucleotides. Luciferase activity of HEK-293 cells transiently co-transfected with the luciferase reporter containing wild-type MGMT-3’UTR or mutant MGMT-3’UTR in the presence of pre-miR-222, miR-221, or scrambled oligonucleotide. Representative of at least three independent experiments. *** p<0.001 versus control, ** p<0,0037 versus control.

We analyzed the sensitivity to TMZ of human glioma cell lines by exposing the cells to 300 µMol TMZ for 48 hours and then assessing cell viability with the MTT assay (Figure 1A). We observed different TMZ sensitivities, which correlated with MGMT levels analyzed by Western blot (Figure 1B). We also observed an inverse correlation between the level of MGMT (Figure 1B) and miR-221 expression in glioma cell lines (Figure 1C). An RNA hybrid alignment bioinformatics search identified a possible binding site for miR-221/222 at position 970 of the 3’ UTR of *MGMT*.

To examine whether miR-221/222 interfered with *MGMT* expression by directly targeting the predicted 3’ UTR region, we cloned this region downstream of a luciferase reporter gene in the pGL3 vector. HEK-293 cells were co-transfected with the reporter plasmid plus the negative control miR (scrambled miR), miR-221, or miR-222. Only transfection of either miR-221 or miR-222 with the wild-type *MGMT*-3’UTR reporter plasmid led to a significant decrease of luciferase activity. On the contrary, co-expression of the scrambled miR had no effect (Figure 1D). In addition, miR-221/222’s effect on the promoter of *MGMT* was reduced with the mutant *MGMT*-3’UTR reporter, in which the seed sequence was mutated. Together, these results demonstrate that miR-221/222 directly target *MGMT*-3’UTR, thereby reducing *MGMT* expression.

### miR-221/222 target MGMT protein and mRNA

**Figure 2:**
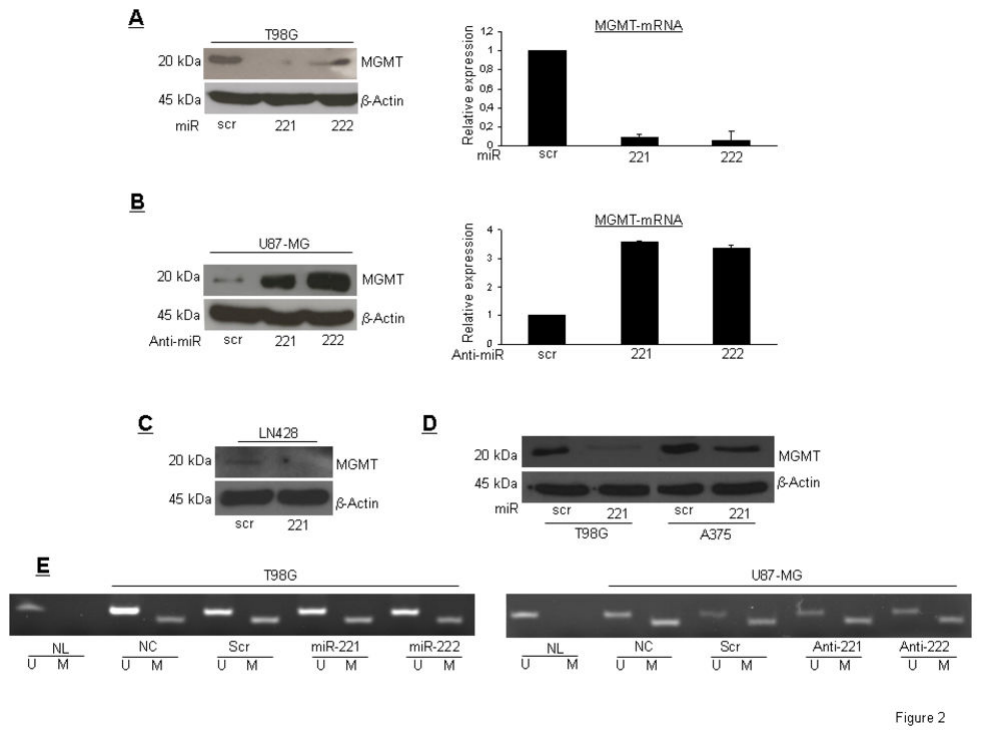
miR-221/222 target *MGMT*. (A) Western blot analysis and real time PCR of MGMT protein and RNA after miR-221/222 transfection of T98G cells. (B) Western blot analysis and real time PCR of MGMT protein and RNA after anti-miR-221 and -222 transfection of U87MG cells. (C) Western blot of MGMT expression upon miR-221 transfection of LN428 cells. (D) Western blot analysis of MGMT expression in T98G cells, as a control, and the melanoma cell line A375 upon miR-221 transfection. (E) Analysis of methylation status of MGMT promoter in T98G and U87MG upon miR- or anti-miR-221/222 transfection. U is for the un-methylated form, M for methylated form, NL is for normal lymphocytes, used as control.

In order to establish a causal link between miR-221/222 and MGMT expression, we transfected T98G cells with either pre-miR-221 or pre-miR-222 for 72 hrs and then analyzed MGMT levels by Western blot and real time-PCR. Upon miR transfection, MGMT protein and mRNA were downregulated (Figure 2A). In contrast, MGMT expression was increased upon transfection with anti-miR-221 or -222 in U87MG cells (Figure 2B). Similarly, miR-221/222, induced downregulation of MGMT in LN428 cells, another TMZ-resistant glioma cell line (Figure 2C), and in A375 cells, a TMZ-resistant melanoma cell line (Figure 2D). Since *MGMT* expression is mainly dependent on the methylation status of its promoter [27], we determined if miR-221/222 acted by modulating *MGMT* promoter methylation. To this end, we performed a bisulfite modification assay by PCR using specific primers for both methylated and unmethylated *MGMT* promoter. As shown in Figure 2E, miR-221/222 expression in T98G cells, or anti-miR expression in U87MG cells, did not modify the methylation profile of the *MGMT* promoter.

### miRs-221/222 modulate TMZ sensitivity in glioma cells

**Figure 3:**
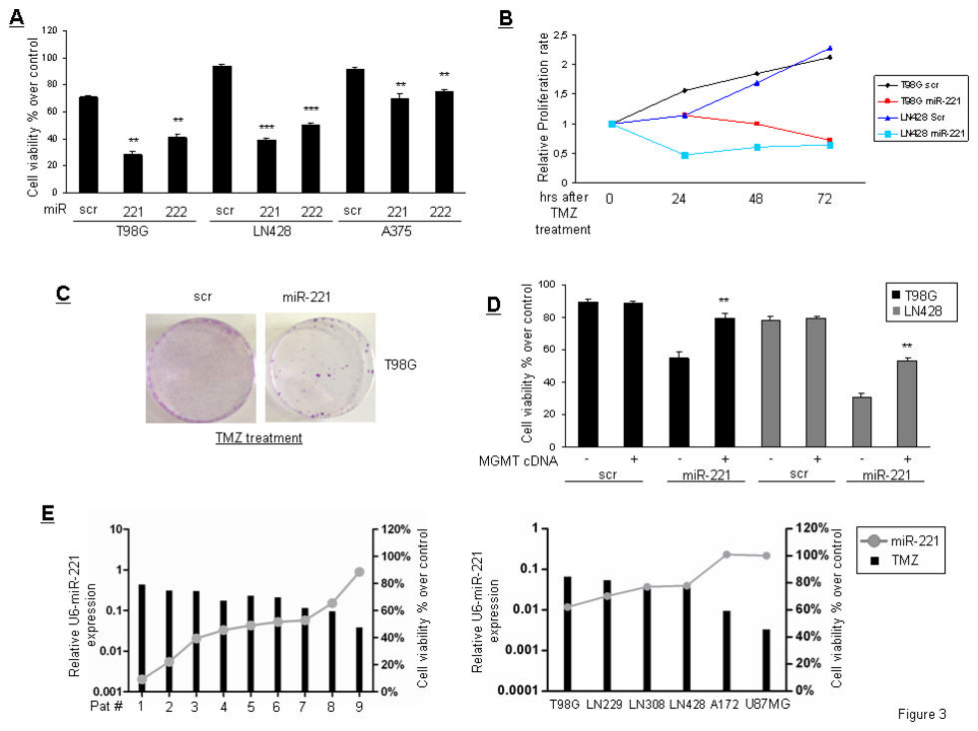
miR-221 modulates TMZ sensitivity. (**A**) Cell viability of T98G, LN428, and A375 cells transfected with miR-221 and miR-222 upon TMZ treatment (300 µMol) for 24 hrs. **p value<0.0082 versus scr column, ***p value<0.005 versus scr column. (**B**) Growth curve of T98G and LN428 cells transfected or not with miR-221 after 24 hrs of treatment with TMZ. (**C**) Colony assay of T98G and LN428 cells transfected with miR-221 and then treated for 24 hrs with TMZ (300 µMol). Cells were left to grow for 6 days after treatment removal. (**D**) MGMT expression rescues cell viability after TMZ treatment in T98G and LN428 cells overexpressing miR-221 **p value<0.0082 versus untransfected MGMT column. (**E**) Correlation between miR-221 expression and TMZ sensitivity in nine primary glioblastoma cell lines and in six glioblastoma cell lines.

To verify if miR-221/222 play a role in the modulation of TMZ sensitivity because of their effects on MGMT expression, we characterized the viability of T98G, LN428, and A375 cells transfected with miR-221/222 and then treated with TMZ for 24 hrs. As shown in Figure 3A, miR-221/222 transfection increased the response to TMZ. These results were also confirmed by proliferation and colony assays (Figure 3B and 3C). To establish a causal link between miR-221 expression and MGMT downregulation, we performed a rescue experiment with simultaneous overexpression of miR-221 and MGMT cDNA in two different cell lines (T98G and LN428). As shown in Figure 3D, the effect of miR-221 on TMZ response was abolished by MGMT overexpression. We then verified in nine different glioblastoma primary cell lines and in six glioma cell lines any correlation between miR-221 expression and TMZ sensitivity. As shown, TMZ sensitivity positively correlated with the expression level of miR-221 (Figure 3E).

### miR-221 promotes apoptotic cell death

**Figure 4:**
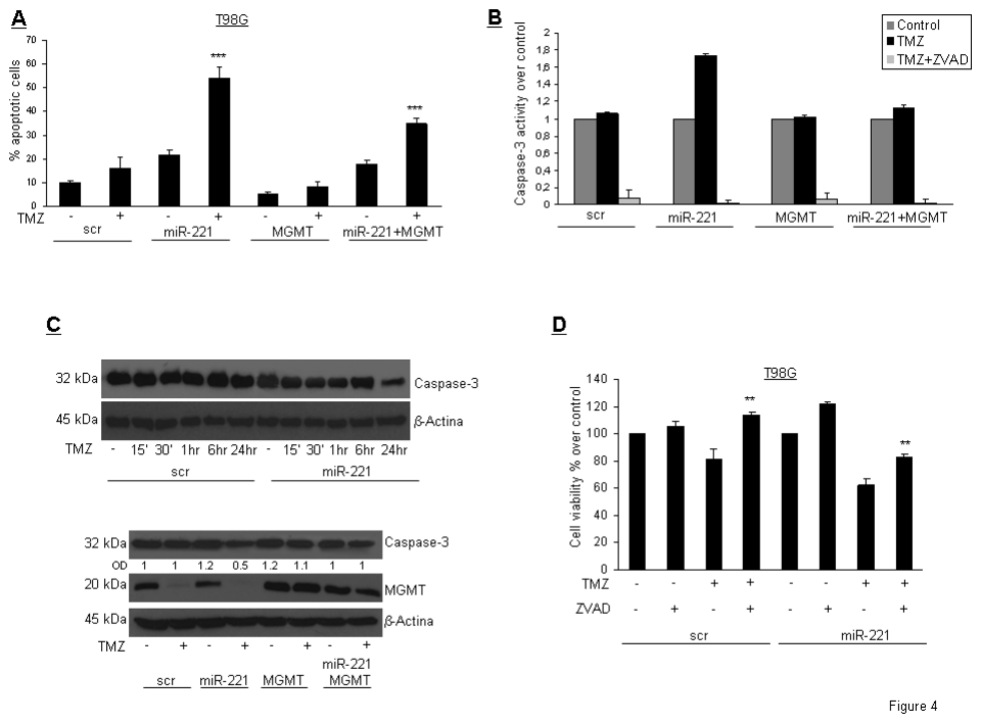
miR-221 promotes DNA damages upon TMZ treatment. (A) Apoptotic cell death assessed by FACS in T98G cells transfected with miR-221 or scrambled sequence and MGMT and treated with TMZ for 24 hrs. *** p value< 0.005 versus untrasfected MGMT column. (B) Active caspase-3 quantification in T98G cells as indicated and treated with TMZ for 24 hrs in the presence or absence of 3 hrs pre-treatment with ZVAD-fmk. (C) Upper panel Time course analysis of caspase-3 activation upon TMZ treatment in T98G cells transfected with miR-221 or with scrambled sequence. Lower panel Western blot analysis of caspase-3 activation after miR-221 and MGMT transfection. (D) Cell viability of T98G cells transfected with miR-221 or with scrambled sequence treated with TMZ for 24 hrs in the presence or absence of 3 hrs pre-treatment with ZVAD-fmk. ** p value< 0.0034 versus only treated TMZ column, Student’s t test.

In order to evaluate the mechanism of TMZ-induced cell death, we assessed the presence of apoptotic cells by PI staining and flow cytometry upon miR-221 transfection and TMZ treatment. We found that TMZ increased apoptotic cell death in miR-221-overexpressing cells compared with control cells. Interestingly, this effect was rescued by the co-expression of MGMT cDNA with miR-221 (Figure 4A). Caspase-3/7 activation assay further confirmed the involvement of the apoptotic machinery. As shown in Figure 4B, miR-221 expression increased caspase-3 activity upon TMZ treatment, while the co-expression of MGMT cDNA with miR-221 abolished this effect. Simultaneous treatment with the caspase inhibitor ZVAD-fmk and TMZ was able to decrease caspase activity, confirming that TMZ induced cell death by a caspase-mediated mechanism. Caspase-3 activation, observed by Western blot in miR-221-transfected cells after 24 hrs of TMZ treatment, was rescued by MGMT cDNA (Figure 4C). Coherently, we observed an increase in cell viability after miR-221 transfection and simultaneous treatment with TMZ and ZVAD-fmk (Figure 4D).

### miR-221 promotes DNA damage after TMZ treatment

**Figure 5:**
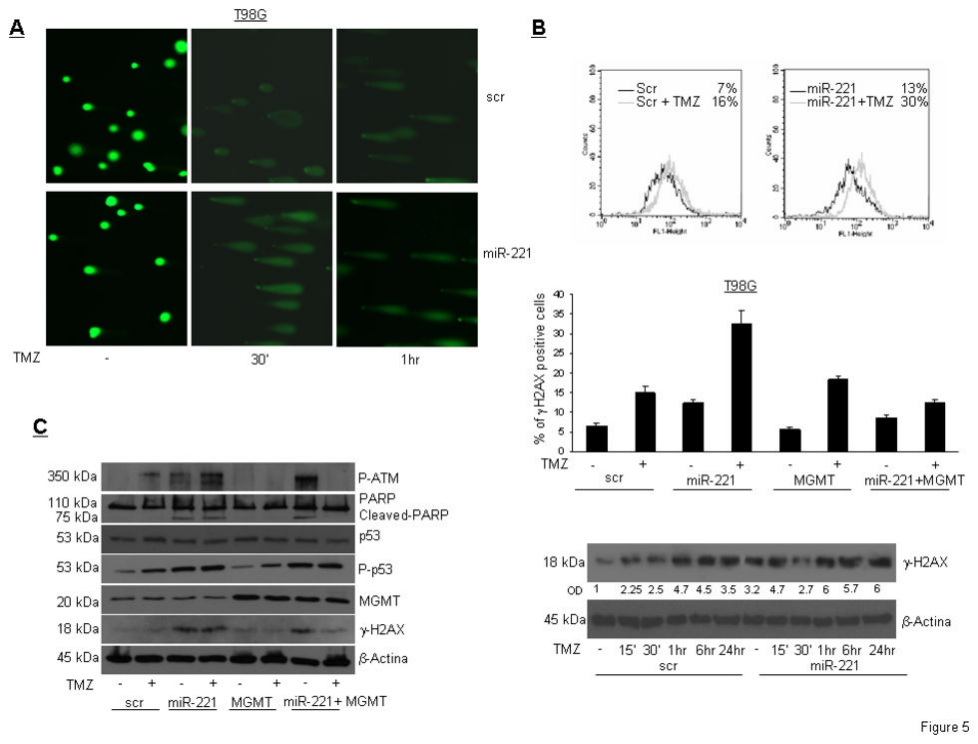
miR-221 promotes DNA damage. (A) Alkaline comet assay of T98G cells transfected with miR-221 and treated with TMZ for the indicated times. (B) Analysis of γH2AX in T98G cells transfected with scrambled control miR or miR-221, treated with TMZ in the presence or in the absence of MGMT cDNA, by immunocytofluorescence (upper and medium panel) or by Western blot (lower panel). (C) Western blot analysis of the indicated proteins upon transfection of T98G cells with miR-221 and MGMT cDNA and TMZ treatment for 24 hrs.

MGMT activity repairs DNA by removing DNA adducts caused by TMZ treatment. The absence of MGMT increases cell death upon exposure to TMZ, but, as a long-term effect, may increase DNA damage, and thus the accumulation of mutations. We investigated whether miR-221 may increase DNA damage upon TMZ treatment by down-modulating MGMT expression. This was assessed by a comet assay, which quantifies double-stranded DNA (dsDNA) breaks, in T98G cells transfected with miR-221 or a scrambled sequence and then treated with TMZ at different times. We found that miR-221 produced a significant enhancement of dsDNA breaks (Figure 5A). To strengthen our hypothesis, we looked for the phosphorylation status of histone H2AX (γH2AX) at Ser139, which reflects dsDNA break formation. As shown in Figure 5B, miR-221 significantly increased γH2AX, as assessed by immunocytofluorescence (upper panel) or by Western blot (lower panel), suggesting that miR overexpression may induce DNA damage. This effect was even stronger in the presence of TMZ, but was rescued by MGMT cDNA (Figure 5B, middle panel). Furthermore, we also observed an increase of other DNA damage markers, such as P-ATM, P-p53^ser15^ and PARP cleavage, upon miR-221 transfection; this was even stronger upon treatment with both miR-221 and TMZ (Figure 5C). These effects were rescued by the simultaneous expression of MGMT with miR-221. Taken together, these data suggest that the targeting of *MGMT* by miR-221 increases DNA damage. This effect was amplified by TMZ treatment.

### MGMT and miR-221 expression in glioblastoma patients

We then evaluated the expression of MGMT and miR-221 in human glioblastoma samples. Patients were clustered into two separate groups: a long survival (survival >15 months) group and a short survival (survival <15 months) group, according to common classification [2].

**Figure 6:**
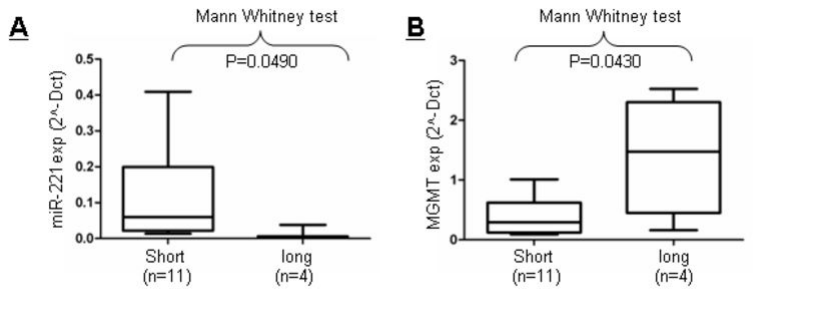
Association of miR-221 and MGMT expression. Mann–Whitney U test analysis was performed to evaluate the association between miR-221 and MGMT expression in long- and short -survival groups of patients. The expression of miR-221 (2^-Dct) (**A**-**B**) and MGMT (2^-Dct) are inversely correlated with patient survival (*p* < 0.0490 and *p* = 0.043, respectively).

We first analyzed the methylation profile of the *MGMT* promoter, and then *MGMT* mRNA and miR-221 levels. We performed methylation-specific PCR (MSP) on 33 human glioblastoma paraffin-embedded tissues, and found 27 to be unmethylated and 4 to be methylated (samples 2, 21, 22, and 28) (Figure S1). For two samples (#31 and #32), it was not possible to define the *MGMT* promoter methylation profile. We then analyzed the effect of miR-221 on *MGMT* regulation among 15 unmethylated samples from which we obtained sufficient RNA for real time PCR analysis. We identified 4 long- (#1, #4, #10, and #14) and 11 short- (#6, #7, #8, #12, #13, #17, #18, #23, #25, #32, and #33) survival patients. We found that the short-survival group exhibited a higher miR-221 level and a lower MGMT level compared with the long-survival group (Figure 6 A,B). These data supports our in vitro evidence of an inverse correlation between miR-221 and MGMT expression. Furthermore, this observation identifies miR-221 as a negative prognostic factor for survival.

## Discussion

Much evidence suggests that the intracellular level of the alkylating enzyme MGMT affects TMZ response in GBM patients [10,11]. Low levels of MGMT are associated with a better TMZ response, because in the absence of MGMT the cells are not able to repair the TMZ-induced base mismatch. Hence, double-strand DNA breaks, DNA mismatch repair, and the apoptotic pathway are activated. *MGMT* expression is regulated by the methylation of its promoter. *MGMT* promoter methylation lowers MGMT levels and accounts for a greater TMZ response when associated with radiotherapy. However, a fraction of patients with unmethylated *MGMT* show some TMZ response, suggesting that promoter methylation is not the only regulatory mechanism of MGMT expression [13,14].

In the present study, we addressed this specific issue by investigating the involvement of miRs in *MGMT* regulation. First, we characterized TMZ sensitivity in a subset of glioblastoma cell lines and primary cells obtained from GBM patients. We found that the analyzed glioblastoma cell lines (T98G, LN428, U87MG, and A172) expressed different levels of miR-221/222 and displayed a consistent difference in MGMT expression. This inverse correlation was also observed in glioblastoma biopsies.

Bioinformatics identified a possible miR-221/222 binding site on *MGMT*. This was confirmed by a luciferase assay and overexpression experiments. The effect of miR-221/222 on MGMT levels was direct and not related to *MGMT* promoter methylation, since miR transfection did not alter the *MGMT* methylation profile. Instead, we found evidence that miR-221/222 regulated MGMT levels, leading to increased TMZ-induced apoptosis, reduced anchorage-independent growth, and reduced cell viability. Overexpression of MGMT cDNA with miR-221/222 rescued the effects on TMZ sensitivity. This result was not restricted to glioma cells, but was obtained also in other cancer cells sensitive to TMZ, such as human malignant melanoma.

It has been demonstrated that *MGMT* may be a target also of other miRs, such as miR-181, in GBM [28]. Zhang et al. demonstrated that miR-181d targets *MGMT* 3’ UTR, and reported an inverse correlation between miR-181d and MGMT levels in human GBM samples, in particular in those samples in which the *MGMT* promoter was unmethylated [28]. However, the modest correlation between miR-181d and MGMT suggested that other miRs may regulate MGMT expression. Therefore, miR-221/222 may be part of this cohort.

MGMT expression may be regulated also thought the p53 pathway. Blough et al. provided evidence that p53 regulates MGMT expression in murine astrocytes, and presented data suggesting that p53 contributes to the regulation of MGMT gene expression in the human astrocytic glioma cell line SF767 [29].

In this manuscript, we demonstrate that miR-221 overexpression increases DNA damage in glioma cells. In fact, miR-221-overexpressing glioma cells exhibited an increase in DNA damage markers, such as P-ATM, P-p53, cleaved PARP, and γH2AX. These markers were activated even in the absence of TMZ, and became increased upon TMZ treatment. MGMT participates in the repair of DNA. Thus, miR-221/222 induces chronic MGMT downregulation, rendering the cells unable to repair DNA damage. It is well established that miR221/222 are oncogenic microRNAs that are upregulated in a number of human tumors [30,31,32]. In GMB tissue and cell lines, upregulated miR-222 and miR-221 expression correlated with the stage of the disease, cell motility, and TRAIL response [19,23,31,33]. We found that miR-221 is a negative prognostic factor, since it is up regulated in short-survival patients and is downregulated in long-survival ones. However, we did not observe the expected correlation between miR-221 expression and response to temozolomide/survival. Arguably, overall survival and therapy response have to be linked to other factors. It therefore seems that the pro-oncogenic effect of miR-221 is more powerful than its potentiation of the response to temozolomide.

The role of MGMT in DNA damage repair has been investigated also in animal models. Reduced expression of this repair enzyme has been thought to result in a spontaneous ‘mutator’ phenotype and to promote neoplastic lesions in the presence of either endogenous or exogenous sources of alkylation stress. Sakumi, et al. showed that Mgmt−/− mice develop thymic lymphomas and lung adenomas to a greater extent when exposed to methylnitrosourea (MNU), suggesting that the DNA repair methyltransferase protected these mice from MNU-induced tumorigenesis [34]. Sandercock et al. reported that MGMT-deficient cells exhibited an increased mutational burden, but only following exposure to specific environmental mutagens [35]. Takagi et al. demonstrated that mice with mutations in *Mgmt* as well as in the DNA mismatch repair gene *Mlh1* developed numerous tumors after being administered MNU. When exposed to a sub-lethal dose of MNU (1mM), the mutation frequency in Mgmt^−/−^/Mlh1^−/−^ cells was up to 12 times that of untreated cells; this effect was not present in control mice [36]. Walter et al. generated transgenic mice overexpressing MGMT in brain and liver, or in lung [37]. They found that expression of the transgene correlated with a reduced prevalence of MNU-induced tumors in liver and in lung and also with reduced spontaneous hepatocellular carcinoma. Reese et al. found that overexpression of MGMT decreased the incidence and increased the latency of thymic lymphoma induction in mice with both heterozygous and wild type p53 alleles [38]. This protective effect was described also by Allay et al., who reported that the incidence of lymphomas was much lower in MGMT transgenic mice compared with controls [39]. Those studies thus suggest that MGMT, other than being involved in the response to therapy, is also involved in DNA repair. Therefore, its inactivation may produce devastating effects on DNA integrity.

In summary, we have provided evidence of the existence of an adjunct mechanism of MGMT regulation, besides promoter methylation, involving miR targeting its 3’ UTR. We have also shown that overexpression of miR-221/222 produces an increase in sensitivity to TMZ via a reduction in the level of MGMT. On the other hand, these miRs increase DNA damage, conferring oncogenic features to glioma cells. This may link miR-221/222 to poor GBM prognosis.

## Supporting Information

Figure S1 **Methylation-specific PCR analyses for MGMT methylation in glioblastoma human tumors.** 33 glioblastoma samples were used for analysis. The SW48 cell line and *in* *vitro* methylated DNA (IVD) are shown as a positive control for methylation, normal lymphocytes (NL) as a negative control for methylation, and water (H2O) as a negative PCR control. U and M indicate the presence of unmethylated or methylated MGMT, respectively. Red colour is for methylated samples, green for unmethylated and orange for undetermined samples.
(TIF)
