# Supplementary figures and images for: miR-221/222 Target the DNA Methyltransferase MGMT in Glioma Cells
Source: PLoS One. 2013 Sep 19;8(9):e74466. doi: 10.1371/journal.pone.0074466 (PMC3798259; doi:10.1371/journal.pone.0074466)

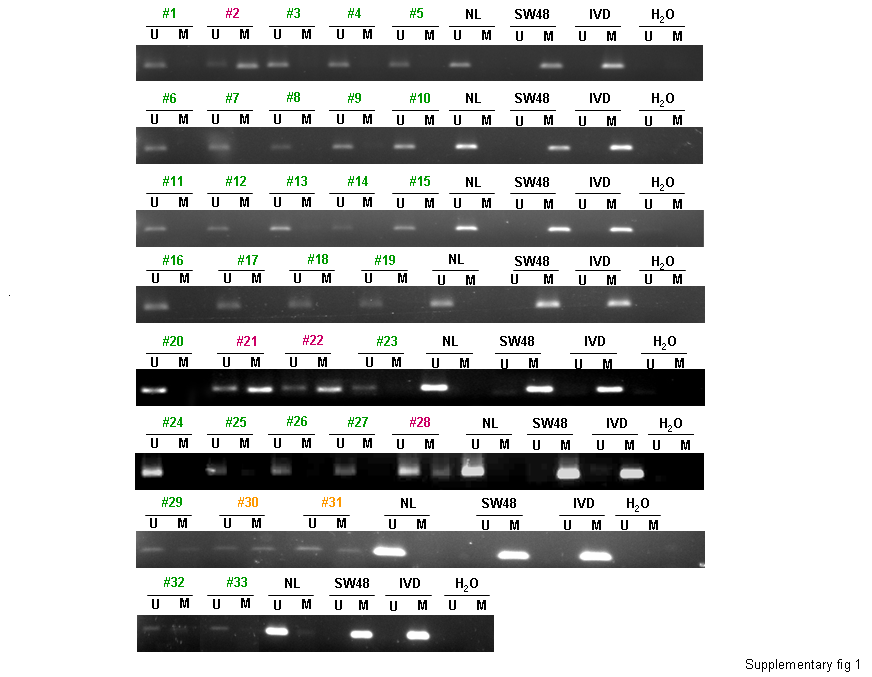

Supplement: Figure S1 — Methylation-specific PCR analyses for MGMT methylation in glioblastoma human tumors. 33 glioblastoma samples were used for analysis. The SW48 cell line and in vitro methylated DNA (IVD) are shown as a positive control for methylation, normal lymphocytes (NL) as a negative control for methylation, and water (H2O) as a negative PCR control. U and M indicate the presence of unmethylated or methylated MGMT, respectively. Red colour is for methylated samples, green for unmethylated and orange for undetermined samples. (TIF) [file pone.0074466.s001.tif]
